# An office building outbreak: the changing epidemiology of tuberculosis in Shenzhen, China

**DOI:** 10.1017/S0950268820000552

**Published:** 2020-02-24

**Authors:** X. J. Guo, H. E. Takiff, J. Wang, G. Y. Han, Y. Z. Fan, G. H. Wu, J. P. Ma, S. Y. Liu

**Affiliations:** 1 Department of Tuberculosis Control and Prevention, Shenzhen Nanshan Centre for Chronic Disease Control, Shenzhen, China; 2 Pathogenomique Mycobacterienne Integree, Institut Pasteur, Paris, France; 3 Instituto Venezolano de Investigaciones Cientificas, Caracas, Venezuela

**Keywords:** Contact investigation, office building, outbreaks, tuberculosis, whole genome sequencing

## Abstract

Tuberculosis (TB) is generally considered a disease that principally afflicts the low-income segments of a population. In the Nanshan District of Shenzhen, China, with the economic transformation and a new Headquarters Economy (HE) emerging, there are now more cases in office workers than in manufacturing workers. To illustrate this trend, we describe a small TB outbreak in an office building located in the centre of the rapidly growing HE district. Two active pulmonary tuberculosis cases were found in workers who shared an office, and whole genome sequencing showed that the genetic distance between the strains of the two cases was just one single nucleotide polymorphism, consistent with intra-office transmission. Investigation of 30 other workers in the same or adjacent offices with interviews, interferon-gamma release assays (IGRAs) and chest X-rays, identified one new TB case and latent tuberculosis infection (LTBI) in 40.0% (12/30) of the contacts. The offices were under-ventilated. None of the IGRA positive, asymptomatic contacts agreed to receive treatment for LTBI, presumably due to TB stigma, and over the next 2 years 69.0% (20/29) of the contacts were lost to follow-up. Treatment for LTBI and stigma of TB remain challenges here. Office workers in the HE of rapidly economic developing areas should be targeted with increased vigilance by TB control programmes.

## Introduction

Tuberculosis (TB), an airborne infectious disease commonly spread in overcrowded environments, is generally associated with poverty and low social class [1, 2]. Recent epidemiological studies have found that while the national incidence of TB falls, it often remains high in big cities [3, 4]. In Western cities, this is often attributed to the urban concentration of high risk groups such as people who are homeless, misuse drugs and alcohol, among whom outbreaks and ongoing transmission have been well documented [3]. In China, where these high-risk groups are no more likely to have TB than the total population, TB has been more prominent problem in factory workers, but office buildings have been considered unusual places for TB outbreaks to occur [5].

The Nanshan District of Shenzhen is one of the fastest economically developing areas in China, with 2 000 000 inhabitants and a reported TB incidence rate of 43.94 new cases per 100 000 inhabitants [6]. The impressive growth of Shenzhen over the past 40 years was based primarily on factories and manufacturing, but in more recent years there has been a shift to an emerging ‘Headquarters Economy’ (HE) that involves a policy of industry upgrading to attract more high-tech companies and people who work in offices. HE refers to the clusters of corporate headquarters of multinational companies and banks in an area with a globalised economy and regional economic integration [7]. A spatial-temporal analysis of Nanshan revealed a low-but-rising TB incidence in the regions where high-tech enterprises and office buildings are clustered, and a still high, but declining incidence of TB in the region where labour-intensive factories are located [8]. Among reported TB cases, the proportion of office workers increased remarkably from 6.97% in 2010 to 26.20% in 2018 while the proportion of factory workers decreased from 42.27% to 24.28%.

In each year since 2015, the Nanshan Centre for Chronic Diseases Control (Nanshan CCDC), which is responsible for TB treatment and control in the district, has identified one or two outbreaks in office buildings. With the increase in crowded high-rise office buildings using central air-conditioning, TB outbreaks in these settings will likely become more frequent. Although many TB outbreaks have been described in schools, prisons, homeless shelters and long-term care facilities [9–12], there have been very few reports of outbreaks in office buildings [5]. To illustrate how these outbreaks are handled and the problems involved, this report describes the interventions, investigations and results in a representative TB outbreak in an office building located in the centre of the HE region of Nanshan.

## Methods

### Setting

With an estimated TB incidence of 63 new cases per 100 000 population in 2017 [13], China is among the 22 high TB-burden countries. Shenzhen's rapid economic growth has attracted migrant workers from all over China [14], who have expanded its population from 30 000 in 1979 to over 15 million in 2019. The Nanshan District, in the southwest of Shenzhen, has a population density of 11 061 people per square kilometres and in 2017 had a per capita GDP of US$ 49 015, the highest of all districts or counties in China [15]. In 2017, there were 113 companies who had their headquarters in Nanshan [16].

The Nanshan CCDC is responsible for TB treatment and control in the district. On a weekly basis, the TB programme routinely checks the information of newly diagnosed cases against that of the previous ones using a web-based TB Information Management System (TBIMS) set up in 2005 by the National Center for TB Control and Prevention (NCTB). An investigation is opened when two or more cases are found in the same workplace. On 9 November 2015, it was noted that two pulmonary tuberculosis (PTB) cases (cases 1 and 2) worked in the same company. A telephone interview with the manager of the company revealed that the two patients worked in the same room, meeting the working definition of an outbreak [17]. The office has an area of 131 square metres and 29 employees, and is located in a company headquarter in the centre of a HE region of Nanshan.

### Investigations and interventions

The principle of graded investigation is that tests and interventions are initiated based on the detected epidemiological links, the characteristics of the index case and evidence of a possible outbreak. Because the two identified cases were both smear and culture positive, and there were 10 other employees working in the same room, six measures were implemented from 10 November 2015 to 16 November 2015: isolation of the patients from the workplace; environmental appraisal for the risk of infection; disinfection and sterilisation of the office; further investigation of the patients; screening of the close contacts and confirmation of transmission through genomic typing of the *Mycobacterium tuberculosis* (MTB) isolates from cases 1 and 2.

The exposed and peripheral areas of the office were visually inspected and an air conditioning engineer was consulted to evaluate the risk of a respiratory pathogen spreading through the ventilation system.

Close contacts, defined as individuals who shared an enclosed space with the index case ⩾4 h a week [18], were offered screening that included symptoms review, chest X-ray (CXR) and the T-SPOT.TB interferon-gamma release assay (IGRA, T-SPOT.TB, Oxford Immunotec Ltd., Abingdon, Oxon, UK). If the contacts could produce a sputum sample, it was tested by smear microscopy, culture and Xpert MTB/RIF (Cepheid, Sunnyvale, CA, USA). The close contacts who were negative with the initial IGRA test in November 2016 were tested again in March 2017 with the IGRA QuantiFERON-TB Gold In-Tube test (QFT, QIAGEN, USA). Any contact with a positive result in any of the IGRA tests but no symptoms, abnormal CXR or positive sputum test was deemed a case of latent tuberculosis infection (LTBI).

After a third active PTB case and seven LTBI coworkers were found among the close contacts, additional measures were adopted between 16 November 2015 and 17 January 2018, including expansion of the investigation, education of coworkers, medical supervision, follow-up tests and the recommendation of treatment for LTBI cases with 9 months of daily self-administered isoniazid.

IGRA tests and CXRs were routinely provided free to all contacts who were voluntarily named by the PTB patients when they were diagnosed. However, as is often the case, the PTB patients appeared reluctant to name contacts other than their family members. The investigation was expanded to include casual contacts who visited the office occasionally and workers in other departments on the same floor. It was recommended that all contacts receive medical supervision by a staff member for suspected symptoms, with follow-up CXRs in the 6th, 12th and 24th month after the first screening [19].

### Study procedures

CXRs were taken and read by a radiologist. The QFT and T-SPOT.TB assays were performed as recommended by the manufacturer, using positive cut-off values of ⩾0.35 IU/ml and ⩾6 spots, respectively.

For whole genome sequencing (WGS), a colony from a Löwenstein–Jensen culture was inactivated at 121 °C for 30 min. The genomic DNA was extracted using the OMEGA EZNA Bacterial DNA Kit, and a paired end library with an insertion length of approximately 300 bp was constructed and then sequenced using the Illumina HiSeq X-Ten sequencing platform, with an average depth of 200. After trimming with Sickle (https://github.com/ucdavis-bioinformatics/sickle), reads were mapped to the reference genome H37Rv (GenBank: AL123456) with Bowtie [20]. The SAMtools/BCFtools suite was used for single-nucleotide polymorphism (SNP) calling [21], and SNPs in the PE/PPE, PE-PGRS and drug-resistance associated genes were filtered [22] using an in-house Perl script. Maximum parsimony phylogeny trees were constructed with MEGA5.

### Statistical analysis

Information of the PTB cases was collected from TBIMS during surveillance and that of contacts was collected on scene during screening. The database was maintained in Excel 2007 and analysed with SPSS 22.0. The descriptive statistical method was used to analyse the characteristics of contacts. The numbers of cases by active and latent TB and proportions out of all those screened were presented. The dropout rate of the contacts during the 2-year follow-up period was also calculated.

### Ethical approval

As all methods of this study was conducted under the regulations governing case detection and contact tracing, and the outbreak investigation was approved by the local health authority, ethical approval and patient informed consent were not required.

## Results

### Cases

During the investigation of this outbreak we detected three cases of active PTB and one case of presumptive latent TB based on the CXR findings and exposure history (Table 1). Cases 1 and 2 were found when undergoing a regular medical records review, and case 3 was discovered during a screening of close workplace contacts, as part of the contact screening and follow-up tests described below. Case 1, who had symptoms for nearly a year before seeing a doctor, had multiple disseminated bilateral patchy images in the lung parenchyma. Cases 2 and 3 were both colleagues who had worked in the same office with case 1.

**Table 1.** Characteristics of patients of a TB outbreak in an office in Nanshan, Shenzhen (China)

Case	Sex	Age	Symptoms started	Place of exposure	AFB	Culture	Chest X-ray	Date of diagnosis	Diagnosis
1	M	32	Approximately 1 year before diagnosis	Office	+	+	Bilateral parenchyma abnormal	28/10/2015	Laboratory confirmed PTB
2	F	29	18/8/2015	Office	+	+	Right upper lobe abnormal	2/11/2015	Laboratory confirmed PTB
3	F	29	Approximately 1 month before diagnosis	Office	−	−	Left upper lobe abnormal	20/11/2015	Clinical PTB
4	F	29	No symptoms	Home	−	−	Bilateral upper-zone abnormal	Not diagnosed	Suspected PTB

AFB, acid-fast bacilli; PTB, pulmonary tuberculosis.

Case 4, who was married to case 1, was found during the routine investigation of the contacts of case 1, before the office outbreak was discovered. A CXR revealed bilateral non-cavitary abnormalities in the upper fields, but case 4 was asymptomatic and refused to receive further medical care. Based on the CXR and exposure history, case 4 was given a presumptive diagnosis of TB. Only cases 1 and 2 had positive cultures for *M. tuberculosis* that could be genome sequenced for evidence of genomic linkage.

### Environmental appraisal

**Fig. 1.**
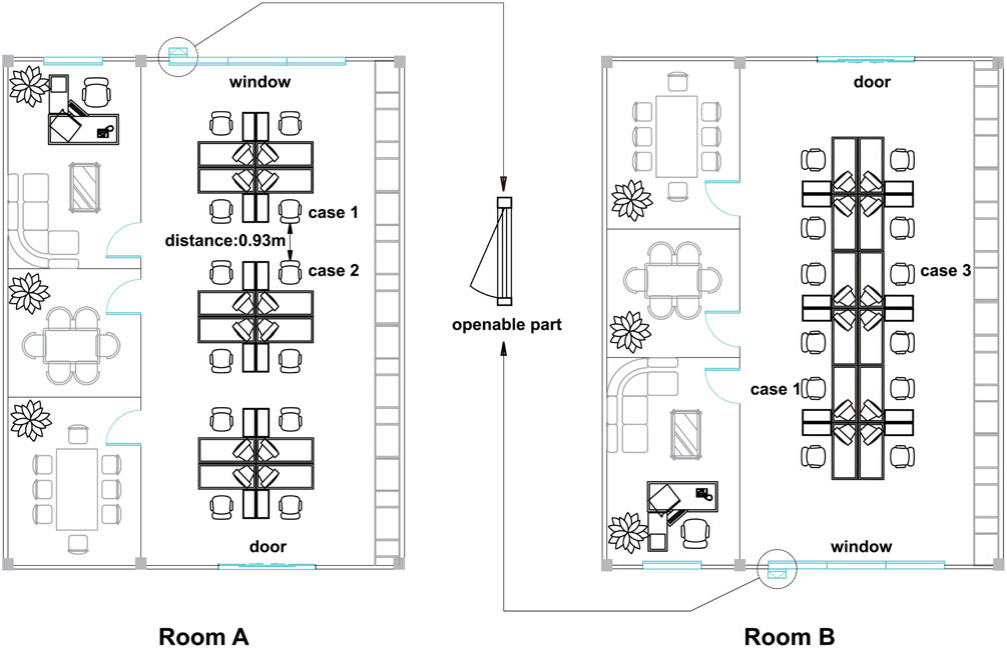
Office rooms as the site of transmission of a TB outbreak in Nanshan, Shenzhen (China). Cases 1 and 2 were seated back to back for about 6 months in room A with a distance between them of less than 1 m. Before that cases 1 and 3 had worked diagonally across the same table for roughly 4 months.

Cases 1 and 2 were seated back to back in room A for about 6 months, separated by less than 1 m, until the outbreak was detected. Prior to this, case 1 had worked in room B at the same table as case 3 for roughly 4 months beginning in January 2015. There was only occasional contact between cases 2 and 3. Room A had an area of 131 m^2^ and a large window, but only a small part of window could be partially opened. Room B was similar to room A (Fig. 1).

Both rooms seemed clean and used a central air-conditioning system for ventilation and air temperature control, but this was seldom used in the spring and autumn seasons. According to the building engineer, each office room and public area inside the building was supplied with fresh air by an exchanger. The indoor air was absorbed into the central air conditioning unit, refrigerated, then discharged back to the room. There was no mechanical air flow between the different office rooms.

### Contact screening and follow-up tests

From interviews with cases 1 and 2 and occupancy rosters provided by their company, a total of 34 contacts were identified, among whom 29 worked at the same site and were close contacts. However, four of these had resigned from the company before the screening and could only be contacted and advised to seek medical attention in a local hospital, leaving 25 close contacts who could be examined. As the investigation expanded, an additional five casual contacts were identified who occasionally visited the office, but the administration of the company prohibited other departments on the same floor from participating in the investigation out of concern for spreading panic. As a result, a total of 30 contacts were screened, who had an average age of 36.6 years (±9.7); 66.7% were males and 76.7% were internal migrants from other cities in China.

**Fig. 2.**
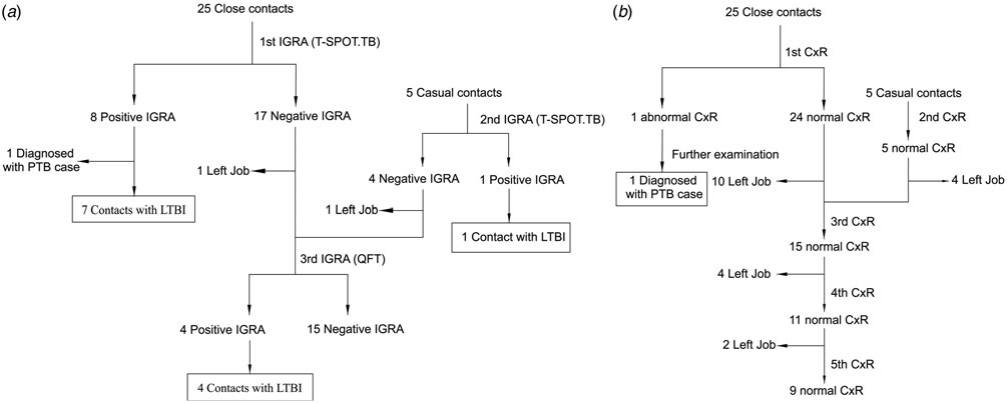
Flow chart for the contact screening and follow-up tests. (a) Screening of contacts for LTBI. (b) Screening of contacts for active PTB. IGRA was conducted thrice for the contact screening and CXR five times for the contact screening and follow-up tests. IGRA T-SPOT.TB was administered on 16 November 2015 and 25 November 2015, and the QFT was performed on 3 March 2016. The first and second CXRs were taken on the same day as the first two IGRA tests, the third on 10 June 2016, the fourth on 19 November 2016, and the fifth on 17 November 2017. IGRA, interferon-gamma release assay; CXR, chest X-ray; LTBI, latent tuberculosis infection; QFT, QuantiFERON-TB Gold In-Tube; PTB, pulmonary tuberculosis.

The contacts were screened three times with IGRAs, the first two times in November of 2015 with the T-SPOT.TB test and the third time in March 2016 with the QFT test. In the first IGRA tests, seven close contacts were positive with the T-SPOT.TB assay and in the second round of IGRAs tests, one casual contact was positive. Four additional close contacts were positive with the QFT test administered in the third round of tests in March of 2016 (Fig. 2a). The eight LTBI cases detected in November 2015 were considered to be prevalent cases while the four LTBI cases whose IGRA tests were negative in November 2015 but positive in March 2016, were considered to be incident cases. Combining all the positive IGRA tests, the prevalence of LTBI was 40.0% (12/30) for all contacts, with 44.0% (11/25) in close contacts and 20.0% (1/5) in casual contacts (Table 2).

**Table 2.** Results of contact screening and follow-up tests

Type of contacts	TB incidence % (*n*/*N*)	LTBI prevalence % (*n*/*N*)	Drop-out rate in follow-up tests % (*n*/*N*)
Close contacts	4.0 (1/25)	44.0 (11/25)	66.7 (16/24^a^)
Casual contacts	0 (0/5)	20.0 (1/5)	80.0 (4/5)
In total	3.3 (1/30)	40.0 (12/30)	69.0 (20/29^b^)

TB, tuberculosis; LTBI, latent tuberculosis infection.

a Of the 25 close contacts, one was diagnosed with TB, and the remaining 24 received follow-up tests.

b Of the 30 contacts, one was diagnosed with TB, and the remaining 29 received follow-up tests.

CXRs were performed five times for the contact screening and follow-up tests. The first two CXRs were taken on the same days that the first two IGRA tests were performed. In the first CXR of contacts performed on 16 November 2015, one more case (case 3) appeared, but this person lived alone and the workmates were already included in the list, so this case didn't contribute more contacts. We attempted to perform a 2-year follow-up of all the contacts, but over this period 69.0% (20/29) of the contacts (not including case 3) left their job and failed to attend follow-up visits. No other abnormal cases were found in the subsequent four CXRs of the contacts who were available for follow-up (Fig. 2b and Table 2).

### Genomic typing and inference with epidemiology

**Fig. 3.**
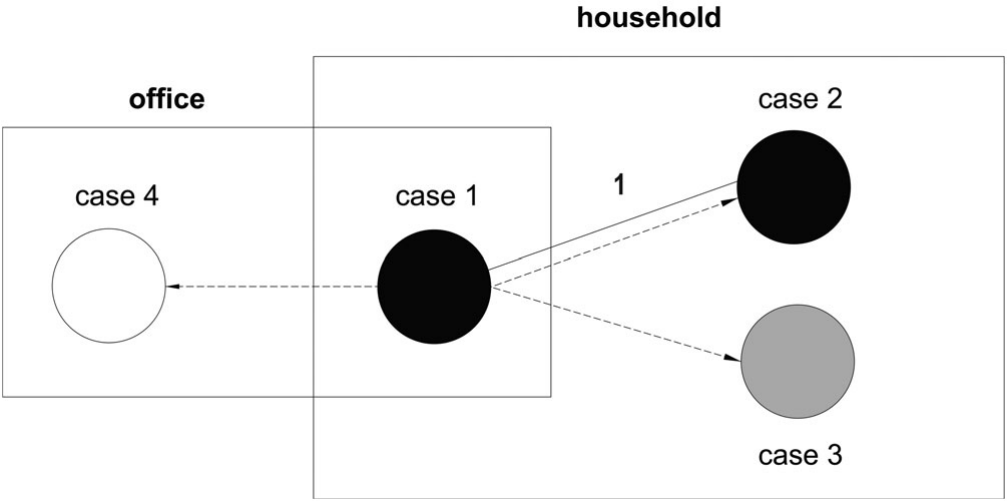
Networks of epidemiological and genomic links of an outbreak in Nanshan, Shenzhen (China). Black circles indicate laboratory confirmed PTB cases, grey circles clinical PTB cases and white circles suspected PTB cases. Solid lines connecting cases represent genomic links with indicated SNP difference on its top, and dashed arrows suggest the likely direction of transmission based on social relationships and onset of symptoms. The boxes indicate principal sites of social contact.

WGS showed that the strains isolated from sputum specimens of cases 1 and 2 belonged to the Beijing family of linage 2 and differed by a SNP. Combining epidemiology and genotype results, the outbreak seemingly comprised four cases: the index case (case 1), two direct secondary cases (cases 2 and 3) presumably transmitted in the office and one presumed infection (case 4) transmitted in the household that was apparently controlled without therapy (Fig. 3). However, because only cases 1 and 2 had isolates for genomic studies, it could not be conclusively proven that cases 3 and 4 were part of the same outbreak.

### Medical supervision

All 12 LTBI contacts were advised to seek treatment in the only two hospitals in Shenzhen offering LTBI treatment, but both resided in other districts of Shenzhen, more than 20 km from Nanshan and none of the contacts sought preventive therapy. No new cases were reported in the 2-year medical supervision of the contacts, but because 69% were lost over this period, the planned medical supervision was incomplete.

## Discussion

Case 1 was characteristic of PTB cases that tend to transmit their infection: they had frequent coughing, a sputum smear that was positive for acid-fast bacilli by microscopy and a long delay before being diagnosed and receiving therapy [17, 23]. The symptoms in case 1 began months long before the onset of symptoms in the other two cases, during which time there was prolonged close contact, and the strain of case 2 was shown to differ by only one SNP from the strain of case 1. Therefore, although there was no isolate from case 3 to test, it seems likely that case 1 was the index case for other two cases in this outbreak.

Shenzhen has a greater TB burden than most Western economically developed cities, and the Nanshan CCDC diagnoses more than 600 TB patients each year, making it impractical to implement active contact investigation on each patient, as is the practice in many European and American cities [17, 23]. IGRA tests and CXR are voluntary and free for contacts named by the patients, but most patients are reluctant to recommend contacts other than their family members. Therefore, it is necessary to review the medical records to identify outbreaks and find clues to the transmission patterns.

The contact investigation we employed used a grading principle that is similar to the concentric circles technique [24], but includes additional aspects, such as treatment for LTBI and follow-up tests. It coordinates the investigations and interventions in an efficient manner tailored to the specifics of the outbreak. The IGRA tests were used instead of the tuberculin skin test to exclude the influence of Bacille Calmette–Guérin vaccination or non-tuberculous mycobacteria [25] and contacts were tested with both IGRAs, T-SPOT.TB and QFT IGRAs, to avoid possible false-negative results when either method is used alone. Since the window period for IGRAs to become positive after exposure has not been clearly defined [26], the third IGRA was performed on the contacts after a 3-month interval.

WGS is more precise than older molecular epidemiology techniques for defining genomically linked strains [27]. A difference of five SNPs has been used as a stringent cut-off for genomically linked strains [27], so the one SNP difference between the isolates of cases 1 and 2 strongly suggests that these cases belong to the same transmission chain, a conclusion that is also supported by their prolonged close proximity at the workplace, where ventilation and sunlight were inadequate for dilution and killing of bacterium [28, 29].

In total, one case of active TB and 12 cases of LTBIs were detected through the contact investigation. Although this appears to be a small number of cases, the incidence of TB (3.3%) and prevalence of LTBI (40.0%) were higher than found through most contact tracing in the United States, 1% and 20–30% respectively [30], suggesting a highly infectious index case and a long duration of intense exposure. The eight prevalent LTBI cases could have been infected by case 1, or they might have been positive as a result of previous exposure outside of the workplace. However, there were four incident LTBI cases, which highlight the importance of follow-up in contact investigations for TB. The prevalence of LTBI in casual contacts was 20%, approaching the 17.87% found in studies of migrant workers in Shenzhen [14], so expanding the contact investigation to workers in other departments, which was opposed by the company administration, did not appear warranted. While the outbreak appeared to have been treated effectively, most of the LTBI contacts could not be followed for the full 2 years, the period during which most cases of active disease caused by recent transmission would develop [19]. However, a third CXR, 6 months after the initial CXR, was performed on the majority (86.67%) of the contacts and found no further cases, it seems unlikely that more PTB cases would be detected even if all contacts had been screened with all of the tests.

The study has some limitations. Because of the limited number of cases and contacts who had LTBI, it was not possible to analyse factors that might have predisposed the contacts to being infected with TB. Although it seems highly likely that case 3 was infected with the same strain as cases 1 and 2, the sputum culture from case 3 was negative, so the genetic relationship of their infections could not be proven. Four contacts were positive by QFT only with the third IGRA test, a QFT assay that was performed 3 months after two negative T-SPOT.TB tests. Because we have no knowledge that any of these four developed active disease, and no additional tests were performed, it is difficult to be sure which results were correct, or whether the inconsistent results were due to the window period necessary for an IGRA test to become positive. It is also possible, although less likely, that some of these contacts were infected from another source during the interval between the tests. Finally, 69% of the contacts were lost to follow-up before the end of the planned 2 year medical supervision, so it is possible that, unbeknownst to us, some LTBI contacts could have developed active TB.

Many of the obstacles to TB control evident in this study could be partially attributed to the stigma associated with TB: a long delay before diagnosis and treatment of the index case; less than full cooperation from the patients and contacts; reluctance of TB cases to name workmates for contact investigation; refusal of LTBI contacts to accept treatment and opposition of the company administration to expanding the investigation to include workers in other, adjacent departments, for fear of provoking panic. Most of the workers in this outbreak setting had university degrees, and although it might be assumed that individuals with higher education would regard TB with less stigma [31], our experience suggests that the stigma persists at all levels of society. However, we must also acknowledge that the lack of nearby facilities to provide and monitor treatment made the LTBI individuals less likely to accept it. The WHO has emphasised the need for preventive treatment of LTBI contacts of PTB cases, especially in high- or upper middle-income countries with an estimated TB incidence less than 100 per 100 000 population [32], and perhaps China needs to improve this aspect of its TB control in eligible cities. The increase in the HE economy means that more workers will spend their days in crowded, high-rise office buildings, which could become frequent hot spots for TB transmission, and obstacles for TB control. There appears to be a need for targeted strategies to increase vigilance for the early detection of cases and transmission in these settings. The Nanshan CCDC is currently trying to establish health guidelines targeting companies to: facilitate screening during the recruitment process; implement annual physical examinations of the workers; encourage reporting of potential TB cases and coordination with the CCDC during an investigation of TB outbreaks; and institute preventive measures including worker education and improvements in the workplace ventilation.
